# The Committee's Reply to Sir Cooper Perry's Report

**Published:** 1913-11-01

**Authors:** 


### YORK COUNTY HOSPITAL.

### The Committee's Reply to Sir Cooper Perry's Report.

THE following is the report to the House Committee of the York County Hospital of the Special Committee appointed to consider Sir Cooper Perry's Report, which we take from the *Yorkshire Herald*, which published it in full last week. The reply has been approved by the Committee and the Medical Board.

The Committee have held eleven meetings and have had interviews with all the members of the Honorary Medical and Surgical Staffs, five of whom have formerly been Resident Officers, and with one former Resident Officer who is not on the Honorary Staff, and also with the Matron.

With regard to the matters dealt with at the inquiry before Sir E. C. Perry, the Committee find that there were twenty-six specific charges made by the Residents, and also the one general charge that the House Committee and Medical Board had paid no attention to representations made by the Residents.

The twenty-six specific charges and the findings in respect of them may be divided into three classes :

Two matters disposed of, upon which comment seems unnecessary.

Eleven charges which failed.

Ten charges as to which the Residents were held justified, but which the House Committee and Medical Board had dealt with, and so were vindicated from the charge of having failed to deal with complaints.

Three charges demanding further inquiry.

The two matters disposed of as to which no comment seems to be necessary were :

The technical question of whether the Residents retired or were dismissed, and

The question of the testimonials to the Residents.

### ELEVEN CHARGES WHICH FAILED.

*Alleged Inadequacy of Night Nursing Staff.*—The allegations made were not accepted as proof.

*Case of Surgical Shock.*—The allegation was held to be unsubstantial.

*Improper Washing of a Patient.*—The allegation was held to lack confirmation and substance.

*Slipper Case.*—The Commissioner reported : "We cannot conclude that in this instance responsibility which ought to rest with a sister was attempted to be put upon nurses."

*Inefficiency of a "Special."*—The Commission held that there was no evidence that the special nurse was inefficient.

*Incompetence of Ward Nurse.*—The allegation was held to lack substance.

*Etiquette.*—The Commissioner thought it "unnecessary to pursue the inquiry further."

*Food and Laundry of Residents.*—The Commissioner did not further investigate the subject.

*Accommodation of Residents.*—The Commmissioner found the complaint to be of a very unsubstantial character.

*Treatment of Sick Nurses.*—Held to be (*a*) clearly disproved, (*b*) unsubstantiated, (*c*) trivial character, (*d*) little substance. The specific instances "are not sustained by the evidence."

*Unhappiness of Nursing Staff.*—No evidence was given in support.

### TEN CHARGES PROVED, BUT DEALT WITH BY THE HOUSE COMMITTEE.

*Six Children's Ward Cases.*—These cases were all dealt with by the House Committee, and the report finds that "no undue delay occurred in taking effective measures."

*Inadequacy of Nursing Staff.*—A sub-committee had inquired into this and made recommendations.

*Misconduct of a Sister.*—This had been dealt with, and the sister had left before the ex-Resident's letter was published.

*Negligence in Reporting of Deaths and Mortuary Case.*—It was held by Sir Cooper Perry to be proved that the Residents left him alive in the mortuary while they had lunch, and that finding should dispose of their charge of 'callous ineptitude' against the nursing staff. The House Committee had made new regulations, and done all that was possible to prevent such an occurrence before the ex-Residents' letter was published in THE HOSPITAL magazine.

There remains the general charge, and the most serious one so far as the House Committee and Medical Board are concerned, that :

"Neither the Medical Board nor the House Committee of York County Hospital would take notice of our reports, or even attempt to aid us in remedying the existent and so apparent evils."

The Committee consider that the charge is dispelled by the finding of the Commissioners that :

"This statement is far from accurate, as the evidence has shown; and also by the fact that as to all the charges which were treated seriously by the tribunal, the House Committee and the Medical Board had already taken action—in some cases completely—and in others by investigation, to be followed up by action in due course.'

With regard to the matters for further inquiry, the Committee report as follows :

### DELAY IN TAKING CASES TO OPERATING THEATRE.

The Committee have investigated the allegation of delay in the only case as to which evidence was given, and they find that it was an unimportant occurrence, and that it is inevitable that at times there may be delay. They do not consider it necessary to do more than draw the attention of the staff to the matter.

### THE GRAZE OR BURN CASE.

The Committee find that there was an error of judgment on the part of the Matron in not reporting this case to the Resident in charge, but at the same time they are satisfied that it was not a serious matter.

### MATRON.

With regard to the strained relations between the Residents and the Matron, the Committee agree that the interests of the hospital must suffer from such a condition of affairs. They found that it was a state of things which, though not inevitable, is very commonly found to exist in hospitals. It was undoubtedly accentuated in the present case, but the result of the fuller information obtained by the Committee led them to the conclusion that the fault did by no means lie entirely with the Matron, and that during the time of the late Residents her position was made one of excessive difficulty.

The Committee refer to the report of Drs. Macdonald and Gayner, in which they say :

"The Matron is always difficult to approach on the question of any modification of existing arrangements.

We admit that this attitude is based on a most profound and accurate knowledge of all the work of the hospital, but we would venture to suggest to her that a little more suavity of manner, and a greater readiness to listen to suggestions, would much tend to the efficiency of the hospital."

The Special Committee are of the same opinion, but at the same time they gladly bear testimony to the fact that the Matron has been most conscientious and hardworking in the discharge of her duties, and entirely loyal to the hospital, and that her attitude in many instances was influenced by a desire to economise the pecuniary resources of the institution. They are of opinion that she has overworked herself, and in particular that it was unwise to take only a week's holiday last year instead of the full month to which she was entitled. The Committee also think that the Matron pays more attention than is necessary to minor details, and that it would be to her advantage in her work if she would delegate some of it to her assistants. The Committee recommend that the Matron should have full authority to engage sisters, and should report engagements to the House Committee at their next meeting.

### Alleged Inadequacy of Staff.

The Committee have found that during the time of the late Residents there was an abnormal demand on the nursing staff for "specials," two specific instances being that at one time no less than eight were put on, and in one case a "special" was kept on for three weeks, when, according to the view of the Honorary Surgeon in charge, forty-eight hours would have been sufficient. Such a state of affairs may or may not have been justified, but obviously it imposed a severe strain on the resources at the command of the Matron.

In order to check any unnecessary use of "specials," the Committee recommend the adoption of the following regulations :—

"(1) When an Honorary Medical or Surgical Officer orders a 'special' he shall sign the patient's bed-ticket as authority for the employment of the 'special.'

(2) This signature shall be valid for a period of not more than 48 hours when the 'special' shall be taken off, unless the signature be repeated.

(3) 'Specials' shall not be ordered by the Residents without an endeavour to communicate with the Honorary in charge by telephone or otherwise.

(4) Where it has not been convenient or possible to do this, the Resident ordering a 'special' shall explain the circumstance to the Honorary in charge at his next visit to the hospital and shall obtain his signature on the patient's bed-ticket as confirming the order, such signature to be valid as commanding the 'special's' services for a period of not more than 48 hours, and to be repeated if necessary where the 'special's' services are required for a longer time."

The Committee have considered the report of Drs. Macdonald and Gayner upon the nursing staff, and they are of opinion that, if the present hours of service are maintained, the staff is adequate for all the ordinary needs of the hospital. It is equal to the average of nursing staffs according to the figures given in Burdett's "Hospitals and Charities Annual."

### Nurses and Their Duties.

For cases of emergency there is an arrangement with the Nursing Home which has proved to be sufficient. The report of Drs. Macdonald and Gayner recommended that the Theatre Nurse should be on call for night work, and the Committee understand that arrangements have been made to comply with this.

The general consensus of opinion which the Committee gathered from the Honorary Officers was that the nursing staff is adequate, and that the nursing is on the whole satisfactory.

The Committee consider that it should be a rule that no ward should ever be left without a nurse, but it should be remembered that owing to the sub-division of some of the wards (which in many ways is a great advantage) nurses may apparently be absent when really engaged in other parts of the ward. The large wards of this hospital consist of two main sub-divisions, two side wards, a balcony, as well as ward offices, day room, bathroom and sterilising room, in any of which nurses may be occupied.

The doorways at present being made from the day rooms into the wards proper, will make the nursing easier. The Committee understand, with approval, that an order has been issued that all the nurses should not go to lunch to the day room simultaneously. The Committee also recommend that electric bells should be fixed in all side wards to ring in a position to be settled, probably in the day room.

### Question of Providing a Sick-room for Nurses.

This has been met temporarily by the earmarking of the side ward in Victoria Ward. The provision of adequate permanent accommodation would involve considerable expenditure in the erection of additional buildings, and this is a matter for the House Committee to consider and submit a scheme to the Court of Trustees for sanction to the use of invested funds for the purpose.

### Admission of Residents and Matron to House-Committee Meetings.

The Committee strongly recommend that the House Committee should request the attendance of the Residents and the Matron at their meetings, so that they may have the opportunity of bringing before the Committee any matters they may think necessary.

### Control of Hospital.

The Committee have given consideration to the general question of the control of the hospital with a view to preventing strained relations between the departments, and of dealing promptly and efficiently with any difficulty which may arise; and they consider that there should be some superior authority always available for reference in cases of difficulty, with power to act, and they recommend :

"That during the intervals between the meetings of the House Committee the ultimate control of the hospital shall be vested in a member of the House Committee, to be nominated annually by that Committee, and a member of the Medical Board, to be nominated annually by that Board."

### REBMAN AND CO.

In connection with a paragraph in our issue of October 18, 1913, concerning the business of the medical publishing house of Rebman and Co., Messrs. E. Salaman (solicitors acting in the proposed sale) write to us to point out that particulars of the business and conditions of sale can be obtained from them by anyone who asks for them. We may add that we neither made nor intended to make any suggestion that concealment of matters of any sort was being practised; but as Messrs. Salaman conceive that such a meaning might possibly be attached to the paragraph in question, we are glad to publish this formal disclaimer.

